# Better palliative care for residents of inpatient nursing homes through qualification of nursing staff – a cluster randomised study

**DOI:** 10.1186/s12904-026-02224-8

**Published:** 2026-07-11

**Authors:** Franziska Radicke, Laura Rehner-Stegen, Heiko Krause, Britta Buchhold, Dennis Nonnenberg, Janina Sarah Dombrowski, Wolfgang Hoffmann, Neeltje van den Berg

**Affiliations:** 1 Institute for Community Medicine, University Medicine Greifswald, Ellernholzstr. 1-2, Greifswald, 17489 Germany; 2 Institute for Nursing Science and Interprofessional Learning, University Medicine Greifswald, Walther-Rathenau-Str. 49 a, Greifswald, 17489 Germany; 3 Institute for Medical Psychology, University Medicine Greifswald, Walther-Rathenau-Straße 48, Greifswald, 17475 Germany; 4 Clinic and Polyclinic for Internal Medicine C, University Medicine Greifswald, Sauerbruchstraße, Greifswald, 17475 Germany

**Keywords:** Nursing homes, Palliative care, End of life care, Qualification of nursing staff

## Abstract

**Background:**

In Germany, 80–90% of people require palliative care in the final stages of life. Inpatient care facilities are increasingly becoming the final place of residence for many people, and therefore also often their place of death. Inadequate identification of palliative care needs can result in substandard palliative care in nursing homes, where residents are often admitted to the hospital towards the end of their lives. This study investigated whether a special palliative care qualifications for nursing staff could improve the care for residents in inpatient care facilities.

**Methods:**

A cluster-randomised intervention study was conducted. Nursing staff from the participating care facilities in the intervention group took part in the 40-hour ‘Palliative Care – Multiprofessional’ basic qualification course. The outcomes were the use of specialised outpatient palliative care, and the documentation of palliative-relevant symptoms and measures for residents in the three months prior to their death. Data from the patient records were assessed and statistically analysed.

**Results:**

During the observation period, 119 residents deceased in the 10 participating nursing homes (5 of which were in the intervention group), of whom 42 were in the intervention group and 77 were in the control group. Specialised outpatient palliative care was documented for 19.0% of residents in the intervention group and 9.1% of residents in the control group. Pain was documented in 57.1% of residents in the intervention group versus 49.4% in the control group. This palliative symptom was documented 225 times in total. An appropriate subsequent measure was documented in 150 cases (73.6% in the intervention group versus 61.9% in the control group). Logistic multilevel analysis showed that residents in the intervention group were more likely to receive a measure for a documented pain event (OR 1.804).

**Conclusions:**

The results show that having a qualification in palliative care leads to palliative symptoms being identified more accurately and appropriate measures being implemented more effectively. Using data from real healthcare settings is challenging because there are no overall standards. Nevertheless, the results reflect real healthcare practice. Advanced training in palliative care helps improves the care provided to palliative residents in nursing homes. Trained staff can help to identify the need for care more accurately and ensure that appropriate palliative care is provided.

**Trial registration:**

German Clinical Trials Register, DRKS00020749 (https://drks.de/search/en/trial/DRKS00020749/entails), 7 May 2020.

**Supplementary Information:**

The online version contains supplementary material available at 10.1186/s12904-026-02224-8.

## Background

 When people need care or if they are no longer able to cope with everyday life on their own, they often move into a residential nursing home. The number of people being cared for in nursing homes in Germany is increasing and, as a consequence, an increasing number of people dies in institutions [1]. The proportion of the population dying in a nursing home is one in four in the US [2], the corresponding proportion in Germany is about 20% [3, 4]. Between 60 and 90% of all people need palliative care at the end of their lives [5, 6]. As people in nursing homes are more likely to be seriously or even terminally ill than in the general population, they are more likely to require palliative care [7]. Therefore, nursing homes are an important setting for end-of-life care and, accordingly, palliative care [8]. Due to an ageing society, the demand will increase and this development will continue. The provision of and knowledge about palliative care is therefore essential for nursing homes. However, although palliative care is becoming more common, it is not available by default [9].

Palliative care is intended to enable people with life-limiting illnesses to enjoy the best possible quality of life. It aim is to prevent or alleviate patients’ suffering through preventive measures and appropriate care [10]. It should also facilitate informed decision-making, identifying the goals of care and treatment preferences of patients and their families [11]. As many people in nursing homes often have cognitive impairments or dementia as well as multiple medical conditions, recognising the need for palliative care can be challenging for caregivers and doctors [12]. Various barriers and problems relating to the provision of palliative care in residential care settings have been identified in the literature [9, 13–16]. For instance, the treatment of (chronic) pain, which is sometimes associated with cognitive impairment, may be inadequate [15]. Shortcomings or gaps in the palliative care of nursing home residents have been identified relating to the basic recognition of palliative care needs and limited access to specialist palliative care [2, 7, 16]. Furthermore, palliative care is often of an inadequate the quality and residents are sometimes admitted to hospital unnecessarily at the end of their lives [9, 13, 14]. In recent years, there has been growing international interest in providing palliative care in nursing homes, with the aim of alleviating some of these issues [17].

In most cases, nursing staff provide care for patients in nursing homes, while external general practitioners and medical specialists are available for medical care as required. Specialised outpatient palliative care is intended for patients whose complex medical and nursing needs cannot be adequately met by the basic palliative care provided by nursing home staff or general practitioners. This care should be available to any eligible patient, including nursing home residents [18]. However, studies have shown that this is precisely where bottlenecks can arise, resulting in gaps in care [14, 19]. This may be due to a lack of cooperation between service providers [14]. However, one analysis found that nursing home residents received a lower proportion of specialised outpatient palliative care than a comparable group living at home. This study, which was based on health insurance data and found that the proportion of specialised outpatient palliative care received at home was almost twice as high as that received in the nursing homes [19].

One critical factor contributing to the inadequate palliative care received by nursing home residents is the insufficient number of nursing staff who have undergone specialist training [14]. Training staff is a promising way to improve palliative care in care settings [11]. Studies have found that better-qualified caregivers lead to better identification of care needs and a reduction in palliative care deficits for nursing home residents [2, 16]. We therefore conducted an intervention study to investigate the effect of staff training on the care of nursing home residents in in their final months.

## Methods

### Aim

The study aimed to investigate whether training nursing staff could improve the quality of care provided to palliative care patients in residential nursing homes. Further objectives included analysing the number of general practitioner contacts, the utilisation of specialised outpatient palliative care services, and the identification of palliative care needs.

### Design and setting

In Germany, both basic and specialised palliative care are available in the outpatient sector. Basic outpatient palliative care is provided by general practitioners and outpatient nursing services. In nursing homes, basic palliative care is provided by nursing staff in collaboration with external general practitioners. Specialised outpatient palliative care is provided by specialist multi-professional teams, including nurses and doctors [20, 21]. To receive services from a specialised outpatient palliative care team, a prescription from a general practitioner or hospital doctor is required. With a prescription, these services are also available in nursing homes [21, 22].

Care staff have a crucial function in identifying and addressing the needs of residents as appropriately as possible. They must assess the urgency of each situation and determine the appropriate level of intervention required, while ensuring that residents’ wishes remain the primary consideration. If any (palliative) needs arise that require treatment by a doctor, care staff serve as intermediaries or interpreters for residents who may not be able to express these needs. If the medical (or palliative) treatment provided proves inadequate, care staff are responsible for recognizing this shortfall and requesting further (specialised) medical treatment as needed.

The study presented was a prospective, cluster-randomised, controlled intervention study conducted in nursing homes in north-east Germany. All publicly searchable nursing homes in two districts in Mecklenburg-Western Pomerania were invited to participate in the study. The inclusion criteria were long-term care seven days a week, 24 h a day. Facilities focusing on people with mental disabilities or mental illnesses, as well as those in which staff had undergone palliative care training within the last 12 months (including the basic qualification, a multi-stage training programme for care home staff, a certified training course in palliative care, non-certified training, and other relevant training), were excluded. Facilities focusing on people with mental disabilities or illnesses were excluded because their residents did not fit the target group; these facilities cater for people of all ages and offer very long-term stays. Using the Chi² method to estimate the number of cases (α < 0.05 and a power of 0.80) resulted in a required number of 93 subjects per group, including a small cluster effect (ICC 0.001 and an average of 1.14 deceased residents per nursing home). Based on the average number of residents dying in nursing homes, the required number of nursing homes was *n* = 7 for both the intervention and the control group respectively. A total of 77 nursing homes were invited to participate in the investigation. All nursing homes identified through research on district social services offices’ and local care support centres’ various platforms were invited to participate. Eighteen nursing homes provided positive feedback. Eight of these withdrew their consent or willingness to cooperate, meaning that ultimately, a total of ten nursing homes could be included (all of the remaining ones). Once all the participating nursing homes had been included, they were given random numbers. These were then sorted in descending order. Cluster enrolment and assignment involved allocating the first half to the intervention group and the second half to the control group. Staff at the nursing homes in the intervention group received standardised training, whereas no intervention took place at those in the control group. The observation period after the intervention was six months. As it would be unreasonable to expect either the care staff or the residents to give their consent in advance of the residents’ possible death, all residents of the participating nursing homes were informed about the study and their right to object. The analysis was confirmatory, assuming that the intervention (training) had a positive effect on the quality of care.

This study was conducted in accordance with the CONSORT Extension for Cluster Trials [23].

### Outcomes

For all patients who died during the six-months observation period, the use of basic palliative care by general practitioners was analysed, as was specialised outpatient palliative care during the time period of 3 months before death. Furthermore, we examined symptoms requiring palliative care (pain, nausea, vomiting, dyspnoea, weakness, loss of appetite, tiredness, depressive mood, anxiety) in terms of the total number of respective entries in the patient records, the number of residents affected, and, if possible and measures taken to treat with these symptoms.

### Intervention

The nursing staff received the basic qualification “Palliative Care – Multiprofessional”. This further qualification module in palliative care for qualified nursing staff is a standardised training programme certified by the German Society for Palliative Medicine and carried out by qualified trainers. Core competencies covered include basic components of palliative care and support for seriously ill and dying people and their relatives, such as basic knowledge of pain and symptom treatment, ethical decision-making and care planning, communication and interdisciplinary teamwork and stress management. Detailed information on the training content can be found at https://www.dgpalliativmedizin.de/allgemein/weiterb-multi.html. The training programme lasted a total of 40 hours (five days of eight hours each), mostly in small groups of eight to 15 participants. In addition to contributions from expert speakers, participants exchanged experiences and reflected on examples from nursing home practice. Participants who successfully completed the training received a corresponding certificate. Passing the qualification depended on attendance, active participation, and completing and presenting group work.

A maximum of 6 nurses per nursing home could participate in the training program. If a registered participant did not attend the training, the training leader made repeated enquiries with the nursing home regarding their whereabouts. For the nurses of the nursing home of the intervention group, the training took place before the beginning of the 6-month observation period. The staff of the nursing residences of the control group were given the opportunity to participate in the training after completion of the observation period. The participating nursing homes were located in northeast Germany. The training programme was divided into different modules due to the study design. The schedule was disrupted by the coronavirus pandemic, forcing the temporary cancellation of planned training modules and rescheduling of training modules to a much later date. This affected the entire study design, delaying the start of the observation period and postponing all subsequent steps. The first training sessions began in August 2020, with the final session commencing in October 2021.

In order to improve the traceability and the completeness of the intervention for this study, it was described in accordance with the “Template for Intervention Description and Replication” (TIDieR) standards [24, 25].

### Data collection

The data on the residents’ palliative care needs were collected from the patient records in the nursing homes. The data were assessed in an anonymised form using a digital, ECRF-based data documentation tool (secure and data privacy compliant). The development of this documentation tool was part of the study, and it was designed to transfer unstructured, varied-format entries from residents’ records in a standardised, uniform manner and record them on a resident-specific basis. Data on all residents who died during the six-month observation period were collected retrospectively. For each resident, entries from their patient records for the final three months of their life were reviewed.

The data collection took place from May 2021 up to and including May 2022.

The following basic patient data were recorded: date of birth, sex, date of move in to the nursing home, existing diseases, date of death. Utilization of external health care providers was recorded as separate contacts, each assigned to one of four categories: (1) home visit in the nursing home, (2) visit in the practice of the doctor, (3) telephone contact and (4) unknown type of utilisation.

Data on the following themes were collected with the respective date of service provision.

- Utilization of external health care providers (general practitioners, medical specialists, specialised outpatient palliative care, emergency service, hospitalization). Only contacts for which the physician group was clearly documented were included
- Symptoms requiring palliative care or with a possible relation to the palliative situation of the resident
- Regular medication as well as medication on demand

The documented care-related entries were assigned to the following categories based on the MIDOS (“Minimales Dokumentationssystem”) documentation instrument [26, 27]: pain, nausea, vomiting, dyspnoea, weakness, loss of appetite, tiredness/fatigue, depressive mood, anxiety. If an entry in the patient record referred to more than one symptom, all symptoms were assigned to the corresponding categories. Furthermore, it was recorded when any measure related to the symptom was documented. In the case of pain, the measures could be categorised more precisely. These included (1) on-demand medication was administered, (2) medical advice was sought from a doctor and (3) other subsequent actions were taken to relieve pain. As with the other symptoms, multiple actions could be documented in response to a single pain event.

### Data analysis

Primary care physicians should provide basic healthcare and counselling for the seriously ill at every visit. These are fundamental components of palliative care. The utilisation of these services (basic palliative care) through contact with the general practitioner and the specialised outpatient palliative care service was analysed descriptively in terms of the total number of visits and frequency per resident. To this purpose, the utilisation of all necessary basic palliative care services was operationalised by the number of contacts with the general practitioners, as the reason for the visits was sometimes not documented specifically.

Care-related entries of symptoms and measures taken from the patient records were analysed descriptively.

For pain, the odds ratio of receiving a measure after the documentation of this symptom, was analysed using a multilevel logistic model. Specifically, the number of measures following documented pain events was compared between the intervention and the control groups using a multi-level analysis. The first level was the individual resident, and the second level was the nursing home level (documented measure at a pain entry nested per resident and the residents were nested in the nursing homes). The model was adjusted for age (continuous variable) and sex, and 95% confidence intervals (CI) around the odds ratios (OR) with standard errors (SE) and p-values were calculated.

The data analyses were performed with Stata^®^ Statistical Software, Release 17.0, StataCorp 2021.

## Results

### Nursing homes

10 nursing homes participated in the study. Of these, five were randomly assigned to the intervention and the control group, respectively. The number of residents in the respective nursing homes, operationalised by the number of beds, varied considerably between the nursing homes. intervention group homes were smaller (median number of beds in intervention group: 72, median number of beds in control group: 112).

16 nurses from 5 nursing homes in the intervention group received the palliative care training. Additionally, 30 nurses from nursing homes in the control group received the qualification after the end of the observation period.

### Residents

The IG had 312 beds in total and the CG had 489. During the observation period, 119 residents died in the participating nursing homes, making up the studied sample of deceased residents. Of these, 42 were from nursing homes in the intervention group. Their characteristics are shown in Table 1.

**Table 1:** Characteristics of residents that deceased during the observation period

	IG		CG		*p*-value
	*N*	%	*N*	%	
Total number of deceased residents	42		77		
Thereof Female	30	71.4	51	66.2	0.561^a^

		SD		SD	
Age at move-in, mean in years	85.1	8.1	84.6	7.1	0.750^b^
Age at death, mean in years	88.3	8.0	87.0	7.0	0.402^b^
Length of stay, mean in years	3.2	2.4	2.2	2.4	0.006^c^

*N* Number, *SD* Standard deviation. ^a^ Chi-square test. ^b^ Welch t-test. ^c^ Mann-Whitney U test. *IG* Intervention group, *CG* Control group

The participating nursing home residents (i.e. those deceased) in the intervention and control groups were comparable in terms of age at move-in and age at death. However, the average length of stay differed significantly: residents in the intervention group stayed for an additional year.

### General practitioner contacts and utilization of specialised outpatient palliative care

**Fig. 1:**
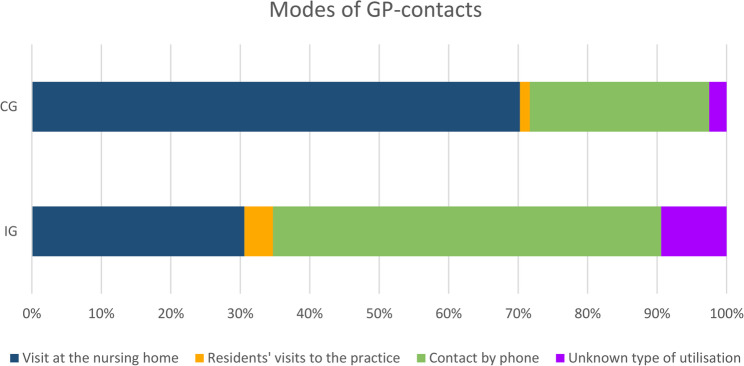
Percentage of contact types with general practitioners by group

A total of n = 449 general practitioner contacts were documented in the patient’ records. More than 80% of residents in both groups had at least one contact with a general practitioner during the observation period. As illustrated in Fig. 1, the various modes of general practitioner-resident contact between IG and CG are represented.

In the intervention group most contacts were by telephone (55.9%; *N* = 95). Most of the contacts (70.3%; *N* = 196) in the control group were home visits in the nursing homes. Resident’s visits to the practices played only a minor role in both groups (intervention group 4.1%; control group 1.4%).

The total number of visits by at least one member of the specialised outpatient palliative care (SOPC) team was *n* = 54. In the intervention group, 19.0% (*N* = 8) of residents had at least one contact with the specialised outpatient palliative care team, while 9.1% (*N* = 7) in the control group had similar contact. The average number of visits per resident receiving at least one SOPC visit was 4.7 in the intervention group and 5.5 in the control group (Table 2).

**Table 2:** Utilisation of general practitioners and specialised outpatient palliative care

Contact to General Practitioner	IG		CG		*p*-value	total	
	*N*	%	*N*	%		*N*	%
Total number of contacts	170		279			449	100.0
Thereof: Visit at the nursing home	52	30.6	196	70.3	< 0.001^d^	248	55.2
Residents’ visits to the practice	7	4.1	4	1.4	0.112^d^	11	2.5
Contact by phone	95	55.9	72	25.8	< 0.001^d^	167	37.2
Unknown type of utilisation	16	9.4	7	2.5	0.002^d^	23	5.1
Number of residents with GP contact	34	81.0	65	84.4	0.629^a^	99	83.2
Number of visits per resident, mean (SD)	5.0	(3.5)	4.2	(3.7)	0.150^c^	4.5	(3.7)
Contacts, days before death: last visit, per resident; mean (SD)	13.8	(19.7)	15.7	(19.9)	0.478^c^	15.0	(19.7)

Specialised outpatient palliative care (SOPC)							
Total number of visits	28		26			54	
Number of residents with SOPC contact	8	19.0	7	9.1	0.118^a^	15	12.6

	N	SD	N	SD		N	SD
Number of visits per SOP-cared resident, mean*	4.7	2.1	5.5	2.5	0.123^c^	5.1	2.3
Visits, days before death: first visit, per resident; mean	21.6	27.1	53.0	48.1	0.246^c^	36.3	40.2
Visits, days before death: last visit, per resident; mean	3.6	4.1	18.6	22.3	0.023^c^	10.6	16.8

*N* Number, *SD* Standard deviation. ^a^ Chi-square test. ^b^ Welch t-test. ^c^ Mann-Whitney U test. ^d^ Fisher’s exact test. *IG* Intervention group, *CG* Control group, *GP* General practitioner

### Identification of palliative care needs and measures by the staff of the nursing homes

A total of 2,114 entries with a possible palliative reference were identified in the care documentation of the 119 residents who died during the observation period. Of these 672 were in the intervention group’s nursing homes and 1,442 were in the control group’s nursing homes. There was high variance in both groups, with the intervention group ranging from 1 to 282 entries and the control group ranging from 116 to 425 entries per nursing home. On average, 16.0 (SD 12.2) and 18.7 (SD 11.0) entries were documented per resident in the intervention and control groups, respectively (*p* = 0.125).

**Fig. 2:**
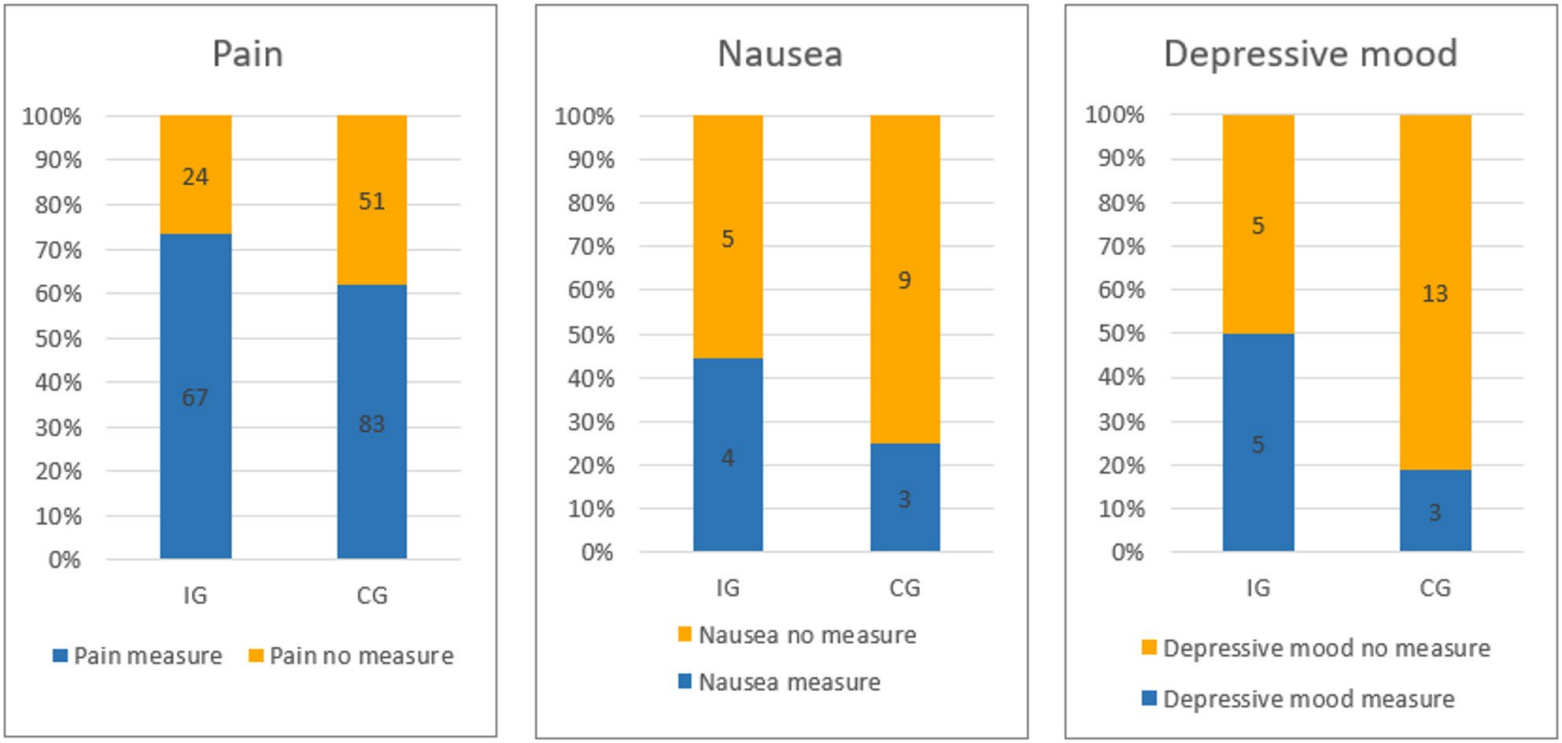
Number of measures for documented pain, nausea and depressive mood entries per group

As can be seen in Table 3, in both groups combined, most entries related to loss of appetite (27%). In the intervention group homes the proportion of entries for this symptom was 10% points higher (intervention group = 33.6%) than in the control group (23.9%). Of the residents in the intervention group homes just under 70% were affected, whereas up to 80% of the residents in control group homes were affect from loss of appetite. Depressive symptoms were documented for one in five residents in the intervention group homes (one in ten in the control group) and a measure was documented in 50% of events (19% in the control group). For pain and nausea, there was also a higher proportion of related documented measures in the intervention group (Fig. 2). Another common symptom was tiredness, which was documented in 60% of intervention group residents and over 70% of control group homes.

**Table 3:** Results of the care documentation entries

Care documentation entry	IG		CG		*p*-value	total	
	*N*	% or (SD)	*N*	% or (SD)		*N*	% or (SD)
Total number of documentations	672		1442			2114	
Number per resident, mean	16	(12.2)	18.7	(11.0)	0.125^c^	17.8	(11.4)
Pain							
Residents with entry^1^	24	57.1	38	49.4	0.416^a^	62	52.1
Entries per resident, mean² (SD)	3.8	(3.5)	3.5	(3.3)	0.751^c^	3.6	(3.4)
Documentation entries³	91	13.5	134	9.3		225	10.6
Measures^4^	67	73.6	83	61.9	0.084^d^	150	66.7
Nausea							
Residents with entry^1^	6	14.3	8	10.4	0.528^a^	14	11.8
Entries per resident, mean² (SD)	1.5	(0.5)	1.5	(0.9)	0.649^c^	1.5	(0.8)
Documentation entries³	9	1.3	12	0.8		21	1.0
Measures^4^	4	44.4	3	25.0	0.397^d^	7	33.3
Vomiting							
Residents with entry^1^	11	26.2	29	37.7	0.206^a^	40	33.6
Entries per resident, mean² (SD)	1.6	(0.9)	2.1	(1.8)	0.457^c^	2.0	(1.6)
Documentation entries³	18	2.7	62	4.3		80	3.8
Measures^4^	8	44.4	30	48.4	0.795^a^	38	47.5
Dyspnoea							
Residents with entry^1^	8	19.0	11	14.3	0.498^a^	19	16.0
Entries per resident, mean² (SD)	2.1	(2.0)	2.7	(2.8)	0.788^c^	2.5	(2.4)
Documentation entries³	17	2.5	30	2.1		47	2.2
Measures^4^	14	82.4	26	86.7	0.692^d^	40	85.1
Weakness							
Residents with entry^1^	13	31.0	31	40.3	0.315^a^	44	37.0
Entries per resident, mean² (SD)	1.8	(1.8)	2.2	(1.4)	0.158^c^	2.1	(1.5)
Documentation entries³	24	3.6	68	4.7		92	4.4
Loss of appetite							
Residents with entry^1^	29	69.0	62	80.5	0.159^a^	91	76.5
Entries per resident, mean² (SD)	7.8	(7.0)	5.6	(5.4)	0.203^c^	6.3	(6.0)
Documentation entries³	226	33.6	345	23.9		571	27.0
Tiredness							
Residents with entry^1^	25	59.5	55	71.4	0.186^a^	80	67.2
Entries per resident, mean² (SD)	4.2	(3.1)	4.3	(3.6)	0.817^c^	4.3	(3.4)
Documentation entries³	104	15.5	238	16.5		342	16.2
Depressive mood							
Residents with entry^1^	8	19.0	9	11.7	0.273^a^	17	14.3
Entries per resident, mean² (SD)	1.3	(0.7)	1.8	(1.3)	0.208^c^	1.5	(1.1)
Documentation entries³	10	1.5	16	1.1		26	1.2
Measures^4^	5	50.0	3	18.8	0.189^d^	8	30.8
Anxiety							
Residents with entry^1^	3	7.1	4	5.2	0.666^d^	7	5.9
Entries per resident, mean² (SD)	1.3	(0.6)	1.3	(0.5)	0.823^c^	1.3	(0.5)
Documentation entries³	4	0.6	5	0.3		9	0.4

*IG* Intervention group, *CG* Control group. ^1^Residents: percentage of residents by group (IG or CG). ²Mean for residents with at least one entry for the corresponding symptom. ³ Documentation entries: percentage of total documentation. ^4^Measures: percentage of entries for the corresponding symptom. N: Number. SD: standard deviation. ^a^ Chi-square test. ^b^ Welch t-test. ^c^ Mann-Whitney U test. ^d^ Fisher’s exact test

As the pain measures were clear and well documented in most cases, a more detailed analysis was performed for this symptom requiring palliative care. This analysis included *n* = 225 documented pain events in 62 residents. A logistic multilevel model with fixed effects was fitted. Compared to the control group, the intervention group showed a higher tendency to receive a measure to alleviate a pain event, though this difference was not statistically significant (OR 1.804; CI 0.793–4.106; SE 0.757; p-value 0.159). There was no association between age or sex and the probability of a documented measure being taken. The intraclass correlation (ICC) of the Random Intercept Model for nursing homes was < 0.001 and for residents-within-nursing home 0.187 at a p-value of 0.0116 (Likelihood-Ratio-Test).

Additionally, a logistic regression as a sensitivity analysis was performed to counteract possible bias due to the small size of the nursing home and the deceased and its results confirmed those of the multilevel analysis, with a slightly lower OR (1.73; CI (0.960–3.097)) for the probability of a documented measurement and a lower p-value (0.068).

## Discussion

### Main findings

The study yields indications that staff training can improve care for nursing home residents. It was found that some palliative symptoms were identified more effectively, and measures were implemented more frequently for one of the observed symptoms.

There was a difference in how general practitioner visits were conducted between the groups. In the intervention group, most of the contacts were conducted remotely by telephone, whereas in the control group, the general practitioner mostly visited the nursing homes. Residents only visited the doctor’s practice in exceptional cases. The proportion of telephone contacts with general practitioners was significantly higher in most intervention group homes than in control group homes. One control group home had no documented telephone contact with a general practitioner at all. The higher number of telephone contacts in the intervention group may be due to the nurses in the nursing homes of the intervention group being more qualified. It is also conceivable that the increased proportion of telephone contacts is linked to the coronavirus pandemic. However, as the relevant regulations applied across the entire district and the participating institutions were located in the same districts, these conditions therefore applied to both groups. Furthermore, personal preferences could also account for the differences. As this was not investigated, it can only be stated that the percentage of telephone contacts was significantly higher in several of the IG institutions than in those of the CG.

The third most common symptom was pain. Unlike many other collected symptoms, it is possible and feasible to take direct countermeasures for pain. However, not every symptom can be addressed, either because it is not possible (e.g. residents reporting a past event with no visible effects) or because it is not useful. Countermeasures for pain were documented more often in the intervention group, though not significantly so. Nevertheless, a clear discrepancy emerges in the percentage of pain events that resulted in a documented measure. This occurred in three out of four cases within the IG facilities, yet it was observed in only three out of five cases within the CG facilities.

Pain is common among residents of nursing homes, with up to 85% of residents reporting pain. Of these, more than half suffer from chronic pain [28]. In our study, the documentation of pain events is approximately equal between the groups. However, the provision of pain medication and/or physician information was proportionally higher in the intervention group than in the control group. Belonging to the intervention group was also associated with a higher (though not significant) probability of taking measures to alleviate pain in the multilevel analysis. About one-fifth of the training programme’s content and time allocation focuses on pain recognition and measures to be taken in palliative care, as pain is a frequently occurring symptom. The participating nursing staff received instruction on the importance of recognising pain and responding appropriately. The findings presented may indicate that this instruction was effectively received by the participants.

In general, little evidence exists to demonstrate the effectiveness of palliative care interventions in residential care settings [11]. The main reason for this is the highly complex research situation, in terms of both the setting and the people involved (residents, relatives, carers and indirectly involved doctors). This high degree of individuality (at the level of both the resident and the nursing home) makes systematic comparisons difficult. The same applies to the present study, in which the complexity of the interplay between setting, residents and data completeness coexists with uncertainties regarding the association between these factors and the measured outcomes.

As with the present study, the results of extant literature on interventions (primarily staff training) generally demonstrate positive changes, albeit in different areas and to varying degrees. The positive effects of staff training have been demonstrated in Australian and Swedish studies [29, 30]. Agar et al. documented these effects in relation to person-centred palliative care [29]. As is evident in the present study, analogous findings (i.e. interventions targeting depressive symptoms) have been reported in other studies. The Swedish study demonstrated a substantial decline in individual symptoms (e.g. shortness of breath and nausea) among care home patients following the implementation of the ‘Liverpool Care Pathway for the Dying Patient’ [30]. Conversely, the present study revealed that the proportion of residents manifesting symptoms of nausea and shortness of breath was marginally elevated in the intervention group (IG), although this could also be attributable to heightened sensitivity. The documented interventions for nausea were proportionally higher in the IG, although the overall numbers were low. Meanwhile, those pertaining to shortness of breath were found to be comparable. In a study by Temkin-Greener et al., a facility-based initiative to establish a team for palliative and geriatric end-of-life care and to train staff did not result in a significant improvement in pain or depressive symptoms. However, the authors observed positive effects on depressive symptoms in the subgroup of care homes that implemented the intervention continuously (six out of 14 homes) [31]. These findings are consistent with those of the present study regarding measures for depressive symptoms and pain.

The only significant discrepancy between the care homes pertained to the duration of residence of deceased residents within the facilities. The mean duration of residence for individuals in the IG was almost double that of those in the CG prior to their demise. The aetiology of this circumstance remains unclear and may be multifactorial in nature. These include higher resident satisfaction and the generally shorter lifespan of CG facilities, given the steadily growing demand for care homes and the frequency with which new ones are established. It is also conceivable that residents in care homes in the CG only moved into the facilities once their illnesses had reached a significantly more advanced stage. These aspects were not investigated and can therefore only be listed here as possible explanations. In one facility, there was only one care documentation entry for a resident who had died there. However, given that other nursing homes were similarly characterised by only a single documentation entry per resident, this home was nevertheless incorporated into the analysis. Affiliation with a rehabilitation clinic renders this home a special case.

Time is one of the most important factors when it comes to providing appropriate care for terminally ill patients. Caregivers verry often have limited resources due to increasing workloads and minimal staffing ratios. The current shortage of qualified staff exacerbates this problem. Overall, these conditions make it difficult to implement training measures in nursing homes. This makes it all the more important to develop networking structures to facilitate better cooperation across the sectors and to involve volunteers, for example. Implementing rules for the qualification of nursing home staff — for example, defining an optimal proportion of staff with a palliative care qualification— could improve the availability and quality of palliative care in nursing homes.

In contrast to the US, where residents only receive palliative care if the disease is expected to lead to death within the next 6 months [7], in Germany the need for palliative care is usually determined by the family doctor and can start much earlier. General practitioner can provide general outpatient palliative care themselves and, if more specialised care is needed, refer the patients to a specialised outpatient palliative care team. It is therefore important for residents of care facilities that their general practitioner recognises their need for palliative care or are informed about this need by the nursing staff. The relationship between general practitioners and nursing home staff is crucial because general practitioners decide whether to request specialised outpatient palliative care. The nursing staff can therefore only identify the resident’s potential care needs; the decision is made by the GP. Nevertheless, this communication of needs between the resident and the GP can be better identified and conveyed by trained and competent nursing staff. Contact with the general practitioner of the resident is only possible during consultation hours. The utilisation of emergency medical services in the event of deteriorating health is often undesirable in end-of-life care.

A higher proportion of residents in the intervention group were treated by a specialised outpatient palliative care team than in the control group. The lower provision of specialised outpatient palliative care among nursing home residents, as described by Rehner et al. [19], could be counteracted by the intervention in the present study. The higher number of in-person visits between GPs and CG home residents may indicate a lower number of SOPC team visits. However, the number of visits is not the decisive criterion, but rather how well GPs recognise and treat palliative needs. Alternatively, it could suggest that residents of care homes experienced symptoms that could not be adequately managed by nursing staff, who therefore consulted GPs more frequently by telephone to better meet residents’ needs. Consequently, the higher number of in-person contacts alone cannot be used to deduce whether this led to a greater or lesser need for PC team visits.

In the German study area, regions are assigned to specific SOPC teams to provide care. However, these regions do not exactly correspond to the base districts covered by this study. This can result in unequal care provision. Discussions with several care home managers revealed that some requests for SOPC care were rejected because the responsible team lacked the capacity to provide it.

As therapeutic measures are often carried out against the will of the patient during an emergency medical response in a palliative care setting, an emergency form has been developed to clearly document their preferences. Developed by the Federal Working Group for Hospice and Palliative Care in Mecklenburg-Western Pomerania, the form records individual wishes and priorities for emergencies, ranging from maximum treatment to purely palliative measures where possible. This ensures that palliative care is provided in accordance with the patient’s wishes, even in an emergency situation.

Ersek et al. advocate incentives for training programmes to include palliative care content, preferably as part of the primary education [15], but also for specific qualifications to raise awareness of the palliative signs and needs of residents among staff who have many years of experience working in care.

### Strength and limitations

As the documentation of symptoms and measures in nursing home patient records is not standardised, and there is no established survey instrument for documenting and measuring palliative care in nursing homes, the present study adopted Cole et al.‘s suggestion and developed a documentation tool that uses routinely collected care data to take into account residents’ personal care needs [32]. Due to our method of data collection, changes over time were also recorded. This is one of the main criteria for outcome measures in palliative and end-of-life care research, as set out in the MORECare guidelines [33].

The small number of participating nursing homes and deceased persons increases the uncertainty surrounding the care documentation. Only documented parameters could be analysed: if a symptom or measure was not documented, it could not be ruled out that it was actually present. However, the conditions for the intervention and control groups were the same, so the groups are comparable. Another possible source of bias is that symptoms (e.g. vomiting or shortness of breath) were sometimes self-reported by the residents. It was therefore not always possible to take action in these cases, as the event in question may have occurred in the past. Furthermore, caregivers only documented information provided by the residents. However, again, the nursing homes in the intervention and control groups nursing home were equally affected. It was not possible to determine how often nurses who had received the qualification were actually involved in the treating of the included patients. In addition, there are structural and situational conditions that may have impacted the studies parameters, but which could not be captured. This includes, for example, the availability of general practitioners and their capacity and possibilities to assess and refer residents to a specialised outpatient palliative care team. The care staff act as intermediaries in this process; they must try to identify the residents’ needs as accurately as possible so that they can communicate these to external healthcare professionals. On the other hand, they themselves can only intervene to a limited extent and cannot make medical decisions. The corona pandemic during the study period significantly impacted the feasibility of the training courses, as well as everyday work and life in the participating nursing homes.

It is possible, that nursing homes that are actively involved in palliative care were more likely to take part in our study. This would imply that they were already aware of the issue and may have already implemented relevant measures. It can therefore be assumed that the standard of palliative care in the participating nursing homes is above average. The quality of care documentation in these nursing homes is also likely to be good, as the staff were aware that their participation would provide access to regular care documentation. However, as this applies equally to both the intervention and control groups, we do not anticipate significant bias in this regard. Nevertheless, it is possible to deduce some general conclusions from this. It is evident that, even in care homes where there is an awareness of palliative care, there is scope for enhancement with regard to the provision of palliative care. Secondly, the findings of this study suggest that staff training would also be beneficial for the residents of these care homes.

The greatest strength of the present analysis is also its greatest limitation: namely, that the care and documentation took place in real, everyday conditions. This meant that the data were not standardised in any way. Consequently, the presence of heterogeneous documentation practices (in terms of both quality and detail) in nursing homes has the potential to result in biased outcomes. Nevertheless, due to the real-life care setting, the documentation is likely to reflect the actual care and culture of the participating nursing facilities.

To enhance the validity of the present study, several steps could be considered: when recruiting, ensure that the facilities between the groups are more comparable (size, location, documentation practices) rather than assigning nursing homes randomly, but instead in matched pairs; keep track of participating nurses within facilities to confirm that the trained staff actually provided care to the deceased residents; ensure better documentation of the interaction between medical (external) and nursing (in-house) staff (who, what, how); for this purpose, it may be necessary to establish documentation standards beforehand.

## Conclusion

In summary, a standardised qualification in palliative care for staff in care homes represents an opportunity to improve the care of residents in the terminal stage. The measures implemented in response to episodes of pain, and in a higher proportion of residents receiving care from specialist palliative care teams in the nursing homes of our IG shows indications for this. Standardised documentation is imperative for the evaluation of the potential effects of interventions that necessitate further validation through additional studies.

## Supplementary Information

Supplementary Material 1.

Supplementary Material 2.

## Data Availability

The data that support the findings of this study are available from the corresponding author upon reasonable request.

## Abbreviations

CI: Confidence interval
OR: Odds ratios
SE: Standard errors
IG: Intervention group
CG: Control group
