# Supplementary material for: Better palliative care for residents of inpatient nursing homes through qualification of nursing staff – a cluster randomised study
Source: BMC Palliat Care. 2026 Jul 11;25:209. doi: 10.1186/s12904-026-02224-8 (PMC13379974; doi:10.1186/s12904-026-02224-8)
Supplement: Supplementary file 1 — Supplementary Material 1. [file 12904_2026_2224_MOESM1_ESM.pptx]

## Slide 1
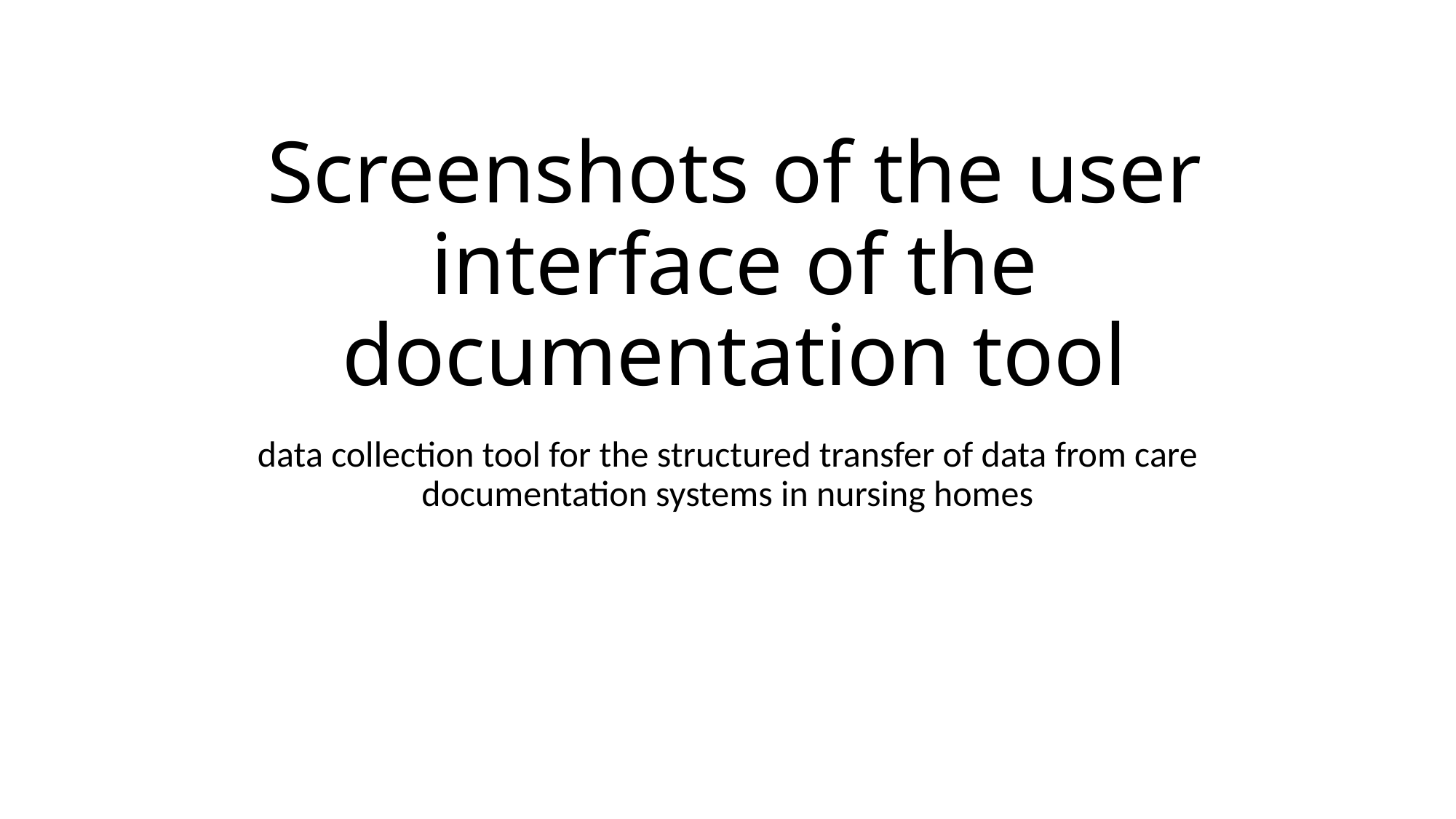

# Screenshots of the user interface of the documentation tool
data collection tool for the structured transfer of data from care documentation systems in nursing homes

## Slide 2
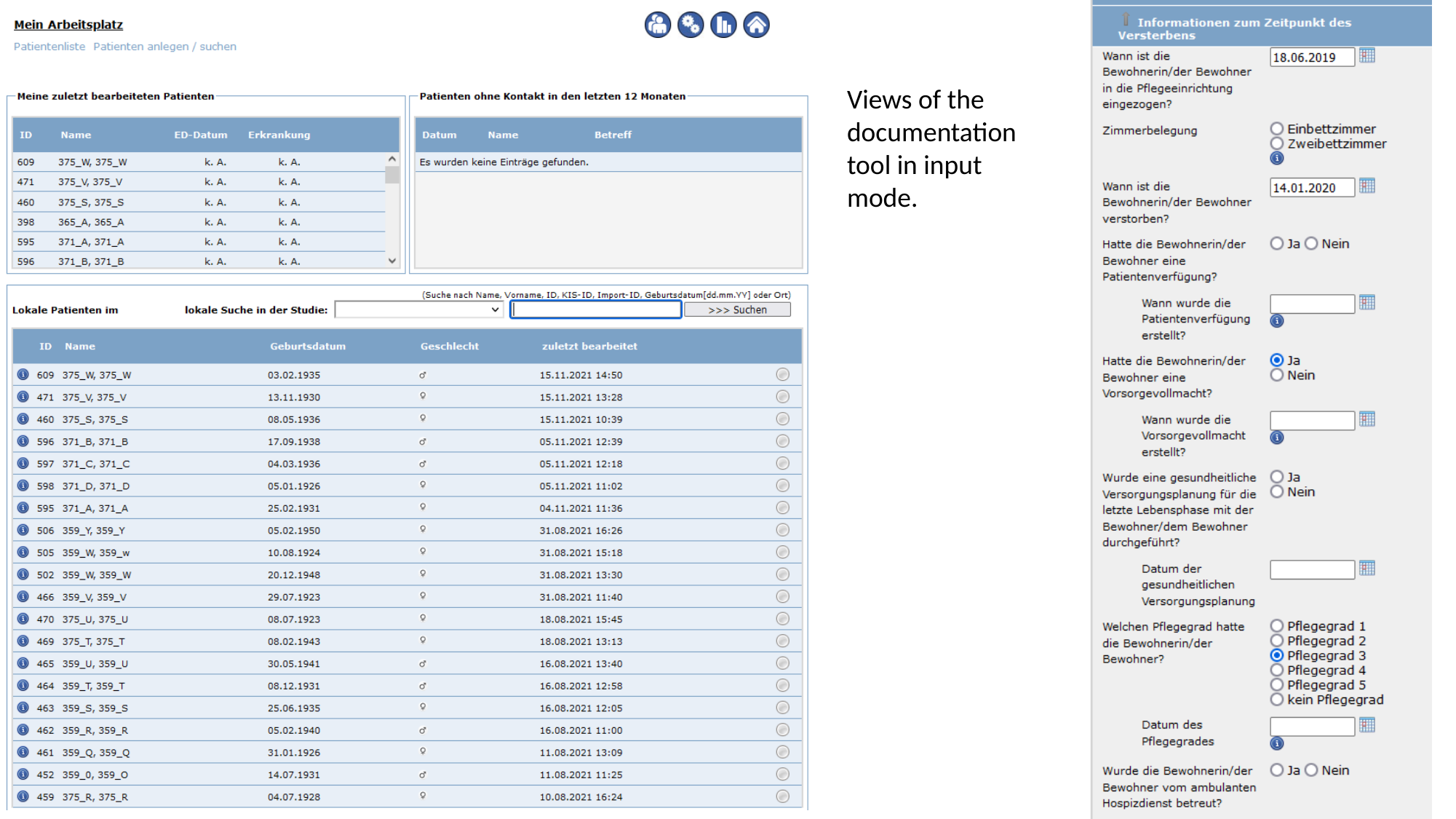

Views of the documentation tool in input mode.

## Slide 3
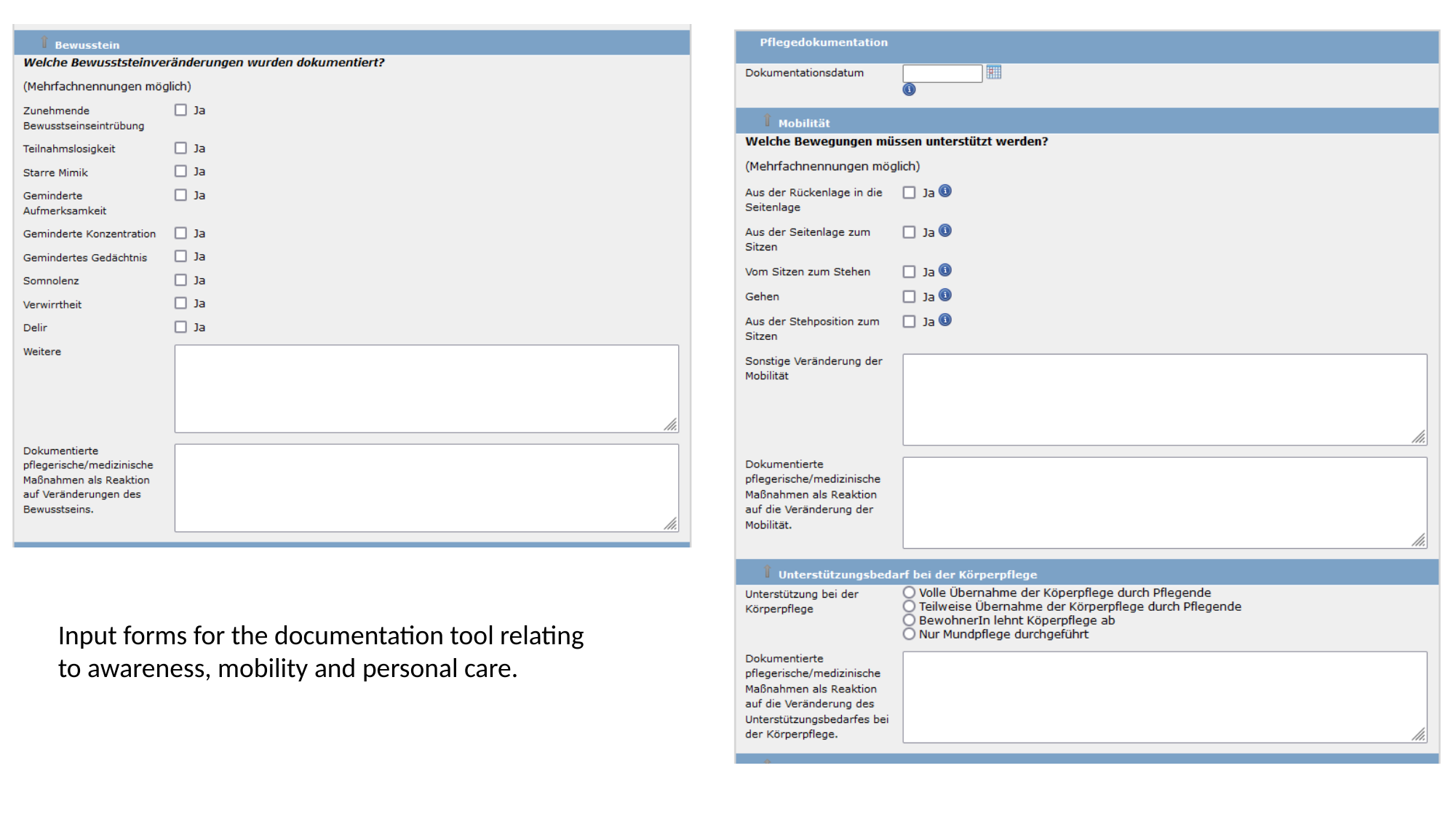

Input forms for the documentation tool relating to awareness, mobility and personal care.
